# Atmospheric Pollution by Fine Particulate Matter (PM2.5) and Low Birth Weight: A Cross‐Sectional Study in Binza Ozone Health Zone, Democratic Republic of the Congo, 2022

**DOI:** 10.1155/jt/7953451

**Published:** 2026-10-26

**Authors:** Christian M. Valuvunina, Maurice M. Ngwimi, Joël Nkiama N. Konde, Guillaume M. Kiyombo

**Affiliations:** ^1^ Department of Environmental Health School of Public Health University of Kinshasa Kinshasa Democratic Republic of the Congo unikin.ac.cd; ^2^ School of Public Health University of Kinshasa Kinshasa Democratic Republic of the Congo unikin.ac.cd

**Keywords:** air pollution, DRC, Kinshasa, low birth weight, PM2.5, quarry

## Abstract

**Background:**

Air pollution is a major risk factor for perinatal health. PM2.5 can cross the placenta and induce oxidative stress and inflammation, promoting fetal growth restriction and low birth weight (LBW). Sub‐Saharan Africa has the highest PM2.5 exposures in the world, but data on perinatal outcomes there are limited. In the DRC, urbanization and unregulated quarries degrade air quality. The Binza Ozone Health Zone in Kinshasa is home to the Carrigrès quarry within densely populated neighborhoods. No study in the DRC has evaluated the link between PM2.5 exposure and LBW. This study aimed to analyze the association between PM2.5 exposure, proximity to the quarry, and LBW.

**Methods:**

Cross‐sectional study of 355 mothers who delivered between January and December 2021 in six healthcare facilities in Binza Ozone. Data were collected via interviews and reviews of antenatal care (ANC) and delivery registries. LBW was defined as a weight < 2500 g. PM2.5 was measured over 48–72 h per health area in September‐October 2022 using calibrated PurpleAir PA‐II‐SD sensors. The PurpleAir PA‐II‐SD sensor is a low‐cost optical particle counter that was calibrated against the Federal Reference Method (FRM) Beta Attenuation Monitor (BAM) in Kinshasa, as described by McFarlane et al. (2021); the calibration equation applied was as follows: PM2.5 (corrected) = 0.52 × PM2.5(raw)+3.2. Sites were classified as “highly polluted” > 75 μg/m^3^ or “polluted” ≤ 75 μg/m^3^ based on the median. Distance to the Carrigrès quarry was categorized as < 500 m and ≥ 500 m. Bivariate analysis was performed using the chi‐square test. Multivariable logistic regression was used to estimate adjusted odds ratios (aORs) and 95% confidence intervals (95% CIs), adjusted for age, parity, education level, maternal alcohol consumption, tobacco exposure (active and passive smoking), nutritional factors (dietary diversity score and mid‐upper arm circumference), and gestational complications. The significance threshold was set at *p* < 0.05.

**Results:**

Median age was 26 years (IQR: 20–36). 67.3% had a secondary education level. LBW prevalence: 12.96%, *n* = 46/355, 95% CI [9.7–16.9]. This prevalence is significantly higher than 7.8% reported for Kinshasa Province in the MICS‐DRC 2017‐2018 survey, confirming the existence of a local aggravating factor. PM2.5 levels ranged from 54.6 to 102.5 μg/m^3^, exceeding both the WHO annual guideline of 5 μg/m^3^ and the WHO 24‐h guideline of 15 μg/m^3^. In bivariate analysis, residing < 500 m from the quarry was associated with LBW: 33.3% versus 9.1%, *p* < 0.001, OR: 4.85 [2.42–9.72]. Gestational complications were also associated: 37.5% versus 10.5%, *p* < 0.001. PM2.5 > 75 μg/m^3^ was not associated: 15.8% versus 11.5%, *p* = 0.312. In multivariable analysis, proximity < 500 m (aOR: 4.12 [1.98–8.57], *p* < 0.001) and gestational complications (aOR: 4.67 [2.11–10.34], *p* < 0.001) remained independently associated with LBW. PM2.5 > 75 μg/m^3^ was no longer significantly associated: aOR: 1.18 [0.59–2.36], *p* = 0.642. Maternal alcohol consumption, tobacco exposure, and nutritional factors were not significantly associated with LBW in this sample (*p* > 0.05 for all).

**Conclusion:**

There is a high prevalence of LBW in Binza Ozone compared to the provincial reference of 7.8%, and an independent association with residing < 500 m from a quarry. Very high PM2.5 levels were not independently associated with LBW, suggesting the effect of a mixture of exposures: coarse particles, noise, and socio–economic factors. These results justify urgent interventions: air quality monitoring, buffer zones around quarries, and the integration of environmental risk assessment into prenatal care.

## 1. Introduction

Air pollution constitutes one of the leading environmental risks to global health, responsible for 7 million premature deaths annually according to the World Health Organization (WHO) [1]. Particulate matter with a diameter of 2.5 μm or less (PM2.5) represents a critical indicator of this pollution due to its ability to penetrate deep into the respiratory tract and cross the placental barrier, inducing oxidative stress and systemic inflammation capable of impairing placental function [2].

Sub‐Saharan Africa exhibits some of the highest PM2.5 exposure levels in the world. The *State of Global Air 2022* report identifies Kinshasa among the 10 most polluted African cities, with annual average concentrations exceeding 40 μg/m^3^, more than eight times the WHO’s annual recommendation of 5 μg/m^3^ [3]. It is important to distinguish between the two WHO guideline values: the annual guideline of 5 μg/m^3^, which represents the lowest concentration at which total cardiopulmonary and lung cancer mortality risk is significantly reduced, and the 24 h guideline of 15 μg/m^3^, which is intended to protect against acute health effects. In this study, all measured concentrations exceeded both thresholds. McFarlane et al. reported an average annual PM2.5 concentration of 43.5 μg/m^3^ in Kinshasa in 2019 [4], confirming the severe degradation of air quality in the Congolese capital. However, the lack of a national surveillance network makes it difficult to accurately assess the exposure of vulnerable populations, particularly pregnant women.

Prenatal exposure to PM2.5 has been associated in numerous studies with adverse pregnancy outcomes, notably low birth weight (LBW), defined as a weight < 2500 g at birth [5]. A European meta‐analysis (the ESCAPE study) demonstrated that a 5 μg/m^3^ increase in PM2.5 exposure during pregnancy was associated with an 18% increased risk of term LBW [6]. The biological mechanisms involve impaired placental vascularization and reduced uteroplacental blood flow, as documented by Van Den Hooven et al. (2012) in the Generation *R* cohort, where PM2.5 exposure was correlated with altered expression of placental nutrient transporters [7].

At the national level, the Demographic and Health Survey (DHS‐DRC 2013–2014) reports a national LBW prevalence of 7% [8]. The Multiple Indicator Cluster Survey (MICS‐DRC 2017–2018) reports a prevalence of 7.8% in the province of Kinshasa [9]. These figures serve as the reference for interpreting the prevalence observed in our study. However, these figures likely underestimate reality due to unregistered home births and limited surveillance in high environmental risk areas.

The Binza Ozone Health Zone (HZ), located in the commune of Ngaliema (Kinshasa), is home to the Carrigrès quarry, an intensive extraction site using explosives for rock crushing, generating significant fine dust emissions. In addition to PM2.5, quarrying activities emit coarser particulate matter (PM10), heavy metals (including lead, cadmium, and arsenic), polycyclic aromatic hydrocarbons (PAHs), and generate noise and vibrations, all of which may contribute to adverse perinatal outcomes. This configuration offers a prime setting to study the impact of residential exposure to PM2.5 on birth weight. This study therefore aims to fill the gap in local data by evaluating the association between atmospheric pollution by PM2.5 and the prevalence of LBW in this heavily industrialized Congolese urban context.

## 2. Materials and Methods

### 2.1. Study Type and Period

This was an analytical cross‐sectional study combining an environmental and a health component. The health component focused on deliveries occurring between January 1 and December 31, 2021. The environmental component, corresponding to air quality measurements, was conducted from September 13 to October 1, 2022. The choice of a time lag was justified by logistical constraints and the hypothesis of stability in the emission sources of the quarries over the considered period.

### 2.2. Study Setting

The study was conducted in the Binza Ozone HZ, located in the commune of Ngaliema in Kinshasa, the capital of the DRC. This HZ is characterized by the presence of the Carrigrès quarry, one of the main rock extraction sites in the city. The area is densely populated, and dwellings are located in immediate proximity to the extraction and crushing sites. Six health areas (HAs) were included: Kinsuka 1, Kinsuka 2, Munganga, Mama Yemo 1, Mama Yemo 2, and Manenga. These HAs were selected because they cover an exposure gradient ranging from immediate proximity to the quarry to more distant areas.

### 2.3. Study Population and Sampling

1. Target population: Mother–newborn pairs residing in the Binza Ozone HZ throughout the pregnancy and who delivered between January 1 and December 31, 2021, in one of the healthcare facilities in the zone.
2. Sample size: The minimum size was calculated using the Schwartz formula, taking an LBW prevalence of 7.8% from the MICS‐DRC 2017–2018, a precision of 5%, and an alpha risk of 5%. The minimum required size was 112 participants. To increase statistical power and limit selection bias, we proceeded with an exhaustive recruitment of all eligible mother–newborn pairs over the study period. In total, 355 mother–child pairs were included.
3. Sampling technique: This was a consecutive exhaustive sample. In each of the 6 healthcare facilities reasonably selected to cover the 6 HAs of the zone, all births occurring between January and December 2021 and meeting the inclusion criteria were consecutively recorded from the delivery registries until the period was exhausted. Mothers were then contacted for the interview and measurement of the distance to their homes.
4. Inclusion criteria: Residing in the Binza Ozone HZ throughout the pregnancy, delivering in one of the 6 healthcare facilities in the zone between January and December 2021, and providing informed consent.
5. Exclusion criteria: Twin pregnancy, newborn with major congenital malformation, and incomplete file.

### 2.4. Selection of Ambient Air Sampling Sites

Six sampling sites were selected, one per included HA. The choice of sites was guided by three criteria: (1) Proximity to the healthcare facilities where health data were collected to maximize the representativeness of the surveyed mothers’ exposure, (2) technical accessibility for the secure installation of sensors on a stable wall or window with access to electricity, and (3) representativeness of the residential microenvironment. The precise geographic coordinates of each site were recorded using a GPS. A posteriori, sites were categorized into two strata based on their average PM2.5 concentration: “highly polluted” if average > 75 μg/m^3^ and “polluted” if average ≤ 75 μg/m^3^. The 75 μg/m^3^ threshold corresponded to the median of the averages of the six sites. The precise geographic coordinates and measurement periods of the six sampling sites are summarized in Table 1.

**TABLE 1:** Ambient air sampling sites with geographic coordinates.

Health area	Site	Address	Latitude	Longitude	Measurement period
Kinsuka Pêcheur 1	CRIDES	Ceinture verte 4	4°20′14.2″S	15°12′52.2″E	13–15 September 2022
Kinsuka Pêcheur 2	CSR Kinsuka 2	Kinsuka 49	4°20′37.2″S	15°13′17.9″E	27–29 September 2022
Munganga	Luyindu	Kimvila 48	4°21′06.5″S	15°13′30.4″E	20–21 September 2022
Mama Yemo 1	Josma	Kembo	4°21′02.0″S	15°13′02.8″E	24–26 September 2022
Mama Yemo 2	Banana	Banana 9A	4°20′54.7″S	15°13′17.9″E	30 September–1 October 2022
Manenga	Siloé	Tembe 12	4°21′49.7″S	15°14′41.8″E	16–19 September 2022

### 2.5. Data Collection

1. Environmental data: PM2.5 concentrations were measured using PurpleAir PA‐II‐SD sensors (PurpleAir, Inc., Draper, Utah, USA). These sensors use laser particle counters to measure optical particle size and count, from which PM2.5 mass concentrations are estimated. The raw data were corrected using the calibration equation developed specifically for Kinshasa by McFarlane et al. (2021), which was derived from collocation with a Federal Reference Method (FRM) Beta Attenuation Monitor (BAM) (Met One Instruments, Inc., Grants Pass, Oregon, USA). The calibration equation applied was as follows: PM2.5 (corrected) = 0.52 × PM2.5 (raw) + 3.2. This calibration is essential to ensure the accuracy of low‐cost sensor measurements in the specific atmospheric conditions of Kinshasa. Each sensor was installed at a height of 2–3 m, sheltered from direct rain, for a duration of 48–72 consecutive hours per site. Concentrations were recorded hourly. Exposure peaks were documented [4].
2. Health data: Data were collected by two trained investigators. For each eligible mother, we conducted a documentary review of the antenatal care (ANC), partogram, and delivery room registries. This information was supplemented by a semistructured interview using a pretested structured questionnaire. This questionnaire was adapted from previously validated instruments used in similar environmental health studies in Sub‐Saharan Africa [10, 11] and was pretested on 20 mothers in a neighboring HA (Boma Bungu) to ensure clarity, cultural appropriateness, and completeness. The internal consistency of the questionnaire was assessed using Cronbach’s alpha (*α* = 0.76). The questionnaire covered age, education level, parity, duration of residence in the neighborhood, estimated distance between the home and the quarry, and the occurrence of pathologies during pregnancy. The questionnaire was expanded to include the following variables: maternal alcohol consumption (yes/no, frequency), tobacco exposure (active smoking: yes/no; passive smoking: yes/no, and exposure at home or workplace), and nutritional factors (dietary diversity score based on 24‐h recall and mid‐upper arm circumference measured in cm).

### 2.6. Variables Studied

1. The dependent variable was birth weight, dichotomized into LBW, defined as a weight < 2500 g, and normal weight ≥ 2500 g, according to the WHO definition.
2. The main independent variables were as follows: (1) The average PM2.5 concentration per site, categorized as > 75 μg/m^3^ (highly polluted) versus ≤ 75 μg/m^3^ (polluted); (2) The distance between the place of residence and the Carrigrès quarry, categorized as < 500 m, 500–999 m, and ≥ 1000 m.
3. Potential confounding variables included the following: maternal age in years, categorized as < 18 years and ≥ 18 years; parity categorized as primiparous and multiparous; education level grouped as none/primary and secondary/university; and the presence of declared gestational complications, namely, hypertension, diabetes, anemia, and malnutrition. Additional confounding variables included the following: maternal alcohol consumption (yes/no), tobacco exposure (active/passive/none), dietary diversity score (low/medium/high), and mid‐upper arm circumference (< 23 cm vs. ≥ 23 cm).

### 2.7. Data Analysis

Data entry was performed in Microsoft Excel 2010 and statistical analysis in SPSS Version 25. Descriptive statistics were presented as medians with interquartile range (IQR) for quantitative variables and percentages for qualitative variables. For bivariate analysis, Pearson’s chi‐square test or Fisher’s exact test, depending on sample sizes, was used to compare LBW proportions between the different exposure categories and maternal characteristics. A multivariable logistic regression was then performed to estimate the independent association between exposures and LBW, adjusting for age, parity, education level, alcohol consumption, tobacco exposure, nutritional factors, and gestational complications. Results are presented as adjusted odds ratios (aORs) with their 95% confidence intervals (95% CIs). The quality of model fit was evaluated using the Hosmer–Lemeshow test. The threshold for statistical significance was set at *p* < 0.05.

### 2.8. Ethical Considerations

The research protocol received approval from the Ethics Committee of the School of Public Health of Kinshasa (ESPK). Administrative authorizations were obtained from the Chief Medical Officer of the Binza Ozone HZ and the Medical Directors of the concerned healthcare facilities. Written informed consent was obtained from all participants after explaining the study objectives. Anonymity and confidentiality of the data were guaranteed. No financial compensation was provided to the participants.

## 3. Results

### 3.1. Sociodemographic Characteristics

A total of 355 mother–child pairs were included in the analysis. The median age of participants was 26 years, with an IQR of 20–36 years. Women under 18 years of age represented 3.4%, *n* = 12. Regarding education level, 67.3%, *n* = 239, of the women had a secondary education, and 14.9%, *n* = 53, had a university‐level education. Only 2.3%, *n* = 8, had no formal education. Regarding marital status, 60.3%, *n* = 214, were married; 34.1%, *n* = 121, were single; and 5.6%, *n* = 20, were in a common‐law union. For obstetric characteristics, 43.1%, *n* = 153, of the women were primiparous. The mean parity was 2.1 ± 1.6. The mean duration of residence in the neighborhood was 6.4 ± 4.2 years. During pregnancy, 17.0%, *n* = 60, of the women reported having presented with at least one pathology. The most frequently reported pathologies were anemia in 8.7%, *n* = 31, of cases, followed by hypertension in 4.8%, *n* = 17, and malnutrition in 2.5%, *n* = 9. Regarding additional risk factors: 5.1% (*n* = 18) reported alcohol consumption during pregnancy; 8.7% (*n* = 31) reported active or passive tobacco exposure; and the mean dietary diversity score was 4.2 ± 1.3 (on a 0–9 scale), with 28.2% (*n* = 100) having a low dietary diversity score (< 4). The mean mid‐upper arm circumference was 26.8 ± 3.1 cm, with 12.4% (*n* = 44) having a value < 23 cm, indicating maternal undernutrition (Table 2).

**TABLE 2:** Sociodemographic characteristics of the respondents (*n* = 355).

Characteristics	*n*	%
*Maternal age in years*		
< 18 years	12	3.4
> 18 years	343	96.6
Median age	26	

*Education level*		
None	8	2.3
Primary	55	15.5
Secondary	239	67.3
University	53	14.9

*Marital status*		
Married	214	60.3
Single	121	34.1
Common law	20	5.6

*Parity*		
Primiparous	153	43.1
Multiparous	202	56.9

*Pathology during pregnancy*		
Yes	60	17.0
No	295	83.0

*Duration of residence in neighborhood (years)*		
Mean ± standard deviation	6.4 ± 4.2	

*Alcohol consumption during pregnancy*		
Yes	18	5.1
No	337	94.9

*Tobacco exposure*		
Active smoker	8	2.3
Passive smoker	23	6.5
No exposure	324	91.3

*Dietary diversity score*		
Low (< 4)	100	28.2
Medium (4–6)	187	52.7
High (> 6)	68	19.2

*Mid-upper arm circumference*		
< 23 cm	44	12.4
≥ 23 cm	311	87.6

### 3.2. Prevalence of LBW

Out of 355 newborns, 46 had a birth weight below 2500 g. The prevalence of LBW was therefore 12.96%, 95% CI: 9.7–16.9. This prevalence is significantly higher than the 7.8% reported for Kinshasa Province in the MICS‐DRC 2017‐2018 survey (*p* < 0.001), indicating the presence of a local aggravating factor in the Binza Ozone HZ. The mean birth weight was 3184 g ± 512 g. The minimum weight recorded was 1100 g, and the maximum weight was 4500 g (Table 3).

**TABLE 3:** Birth weight characteristics of newborns, Binza Ozone HZ, 2021 (*n* = 355).

Parameters	Result
Total count	355
Mean birth weight	3184g ± 512g
Minimum weight	1100g
Maximum weight	4500g
Count of LBW < 2500 g	46
LBW prevalence	12.96%
95% CI	9.7–16.9
Comparison with MICS‐DRC 2017–2018 (7.8%)	*p* < 0.001

### 3.3. PM2.5 Pollution Levels at Sampling Sites

PM2.5 measurements were carried out at the six sites corresponding to the six HAs. Average PM2.5 concentrations over 48–72 h ranged from 54.6 μg/m^3^ to 102.5 μg/m^3^. All sites presented concentrations well above both the WHO annual guideline value of 5 μg/m^3^ and the WHO 24‐h guideline value of 15 μg/m^3^. The Kinsuka 1 site, located less than 500 m from the Carrigrès quarry, recorded the highest concentration at 102.5 μg/m^3^. The Manenga and Mama Yemo 2 sites, the furthest away, presented the lowest concentrations with 54.6 μg/m^3^ and 55.6 μg/m^3^, respectively. The median of the averages of the six sites was 68.3 μg/m^3^. On this basis, we categorized three sites as “highly polluted” > 75 μg/m^3^ and three sites as “polluted” ≤ 75 μg/m^3^ (Table 4).

**TABLE 4:** Distribution by stratum of PM2.5 concentrations μg/m^3^/hour.

Health areas	Sites	Distance to quarry	PM2.5 mean μg/m^3^	Category	Hourly peak
Kinsuka 1	Kinsuka 1	< 500 m	102.5	Highly polluted	04:00–08:00
Kinsuka 2	Kinsuka 2	500–999 m	66.7	Polluted	04:00–08:00
Munganga	Munganga	500–999 m	77.0	Highly polluted	08:00–12:00
Mama Yemo 1	Mama Yemo 1	≥ 1000 m	55.6	Polluted	04:00–08:00
Mama Yemo 2	Mama Yemo 2	≥ 1000 m	70.0	Highly polluted	04:00–08:00
Manenga	Manenga	≥ 1000 m	54.6	Polluted	08:00–12:00
WHO 24‐h guideline value			15		
WHO annual guideline value			5		

The spatial distribution of the 6 sampling sites and PM2.5 concentrations around the Carrigrès quarry in Binza Ozone HZ is presented in Figure 1. The highest PM2.5 concentrations were observed in HAs closest to the quarry, confirming a spatial exposure gradient.

**FIGURE 1:**
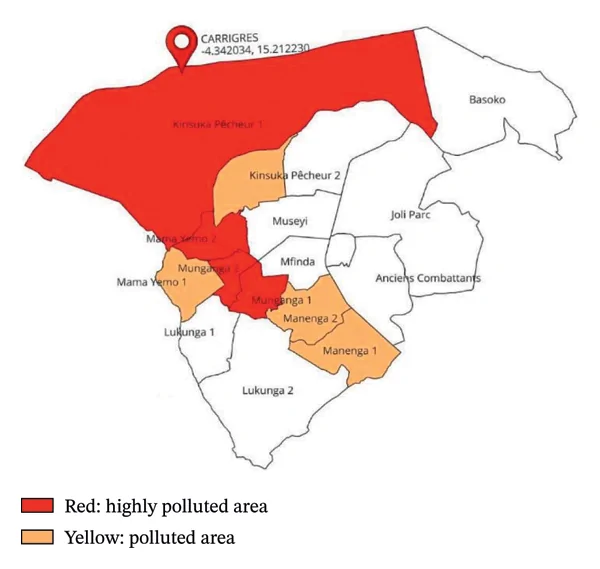
Spatial distribution of the 6 sampling sites and PM2.5 concentrations around the Carrigrès quarry in the Binza Ozone Health Zone.

### 3.4. Bivariate Analysis of Factors Associated With LBW

Table 5 presents the results of the bivariate analysis. Residential proximity to the Carrigrès quarry was strongly associated with LBW. Among women residing less than 500 m from the quarry, 33.33%, *n* = 10/30, delivered a LBW child, compared to 9.06%, *n* = 29/320, for those residing within 500 m or more, *p* < 0.0001. The presence of pathologies during pregnancy was also strongly associated with LBW. Thus, 37.5%, *n* = 22/60, of the women who presented a pathology had a LBW child compared to 10.5%, *n* = 24/295, among those without pathology, *p* < 0.0001.

**TABLE 5:** Prevalence of low birth weight according to characteristics, bivariate analysis (*n* = 355).

Variable	LBW (*n* = 46)	No LBW (*n* = 309)	Total n	% LBW	Crude OR	*p* value
*Distance to quarry*						
< 500 m	10	20	30	33.3	4.85	< 0.001
> 500 m	36	289	325	11.1	1	

*PM2.5 mean*						
> 75 μg/m^3^	18	96	114	15.8	1.45	0.312
≤ 75 μg/m^3^	28	213	241	11.6	1	

*Maternal age (years)*						
< 18	3	9	12	25.0	2.49	0.206
≥ 18	43	300	343	12.5	1	

*Parity*						
Primiparous	24	129	153	15.7	1.51	0.183
Multiparous	22	180	202	10.9	1	

*Education level*						
None/primary	10	53	63	15.9	1.36	0.421
Secondary/university	36	256	292	12.3	1	

*Gestational complications*						
Yes	22	38	60	36.7	4.85	< 0.001
No	24	271	295	8.1	1	

*Alcohol consumption*						
Yes	3	15	18	16.7	1.36	0.812
No	43	294	337	12.8	1	

*Tobacco exposure*						
Yes	5	26	31	16.1	1.32	0.654
No	41	283	324	12.7	1	

*Dietary diversity score*						
Low (< 4)	15	85	100	15.0	1.29	0.531
Medium/High (≥ 4)	31	224	255	12.2	1	

*Mid-upper arm circumference*						
< 23 cm	7	37	44	15.9	1.33	0.423
≥ 23 cm	39	272	311	12.5	1	

Maternal age under 18 years showed a trend toward association with LBW. Indeed, 37.5%, *n* = 3/12, of adolescents under 18 had an LBW compared to 11.8%, *n* = 43/343, for women 18 years and older. However, this difference was not statistically significant, *p* = 0.206, likely due to the small sample size in this category.

Regarding PM2.5 exposure, 15.8%, *n* = 18/114, of women residing in a “highly polluted” area > 75 μg/m^3^ had an LBW compared to 11.5%, *n* = 28/241, for those residing in a “polluted” area ≤ 75 μg/m^3^. This difference was not statistically significant, *p* = 0.312. Education level, parity, and marital status were not significantly associated with LBW in bivariate analysis. Alcohol consumption, tobacco exposure, and nutritional factors (dietary diversity score and mid‐upper arm circumference) were also not significantly associated with LBW in bivariate analysis (*p* > 0.05 for all).

### 3.5. Multivariable Analysis of Factors Associated With LBW

To identify factors independently associated with LBW, we performed a multivariable logistic regression including all variables with *p* < 0.20 in bivariate analysis as well as the main exposure variables. The final model included distance to the quarry, PM2.5 level, maternal age, parity, education level, alcohol consumption, tobacco exposure, nutritional factors, and the presence of gestational complications. The Hosmer–Lemeshow test indicated a good fit of the model to the data, *p* = 0.73, suggesting no significant difference between observed and predicted values.

The results of the multivariable analysis are presented in Table 6.

**TABLE 6:** Multivariable analysis of factors associated with low birth weight (*n* = 355).

	Adjusted OR	95% CI	*p* value
Distance to quarry < 500 m	4.12	[1.98–8.57]	< 0.001
PM2.5 > 75 μg/m^3^	1.18	[0.59–2.36]	0.642
Age < 18 years	2.34	[0.54–10.10]	0.254
Primiparous	1.29	[0.65–2.55]	0.469
Education none/primary	1.08	[0.48–2.43]	0.852
Alcohol consumption	1.22	[0.38–3.91]	0.738
Tobacco exposure	1.15	[0.41–3.22]	0.792
Low dietary diversity	1.31	[0.68–2.52]	0.419
MUAC < 23 cm	1.28	[0.53–3.09]	0.582
Gestational complication (yes)	4.67	[2.11–10.34]	< 0.001
Constant	0.055		< 0.001

After adjustment, only residential proximity of less than 500 m to the quarry and the presence of gestational complications remained significantly associated with LBW. The risk of LBW was multiplied by 4.12 for women residing less than 500 m compared to those residing within 500 m or more, aOR = 4.12; 95% CI: 1.98–8.57; *p* < 0.001. Similarly, the risk was multiplied by 4.67 in the presence of a gestational complication, aOR = 4.67; 95% CI: 2.11–10.34; *p* < 0.001.

On the other hand, exposure to PM2.5 concentrations above 75 μg/m^3^ was not independently associated with LBW, aOR = 1.18; 95% CI: 0.59–2.36; *p* = 0.642. Maternal age < 18 years, alcohol consumption, tobacco exposure, and nutritional factors were also not significantly associated with LBW after adjustment.

## 4. Discussion

This study documents for the first time in the DRC the association between residential exposure near a quarry and LBW in a heavily industrialized urban context. Out of 355 newborns included, 46 had a weight < 2500 g, yielding an LBW prevalence of 12.96% (95% CI: 9.7–16.9). This prevalence is significantly higher than the 7.8% reported at the provincial level in the MICS‐DRC 2017–2018 [9], confirming the existence of a local aggravating factor. The 7.8% figure serves as the reference “normal” prevalence for the Kinshasa Province, against which our results should be interpreted [9]. The mean birth weight was 3184 g ± 512 g, with a minimum of 1100 g and a maximum of 4500 g.

We observed a clear spatial gradient: 33.33% LBW among women residing < 500 m compared to 9.06% at ≥ 500 m, with an adjusted OR of 4.12 [1.98–8.57], *p* < 0.001. This result is consistent with the literature on the effects of proximity to pollution sources [12]. Similarly, Amegah et al. in Ghana documented the impact of domestic and ambient air pollution on adverse pregnancy outcomes, including LBW [10].

A major inconsistency emerges from our results. While proximity to the quarry is strongly associated with LBW, the measured PM2.5 concentrations are not after adjustment: aOR = 1.18 [0.59–2.36], *p* = 0.642. Several hypotheses can explain this divergence. First, the < 500 m distance is likely a marker of multiple unmeasured exposures: coarse PM10 particles, heavy metals (lead, cadmium, and arsenic) that are known to be emitted during quarrying and rock crushing activities and have been associated with adverse birth outcomes, noise, vibrations, stress, and socio–economic precariousness [13]. Regarding the apparent contradiction between the < 500 m distance as a marker of socio–economic precariousness and the absence of correlation with maternal education level, it is important to note that education level is only one dimension of socio–economic status. Other unmeasured factors, such as household income, occupation, housing quality, and access to resources, may better capture the socio–economic vulnerability of women living near the quarry. Furthermore, the lack of association with education level may reflect the homogeneity of our sample, where 82.2% of women had at least a secondary education. Second, our punctual measurements of 48–72 h do not reflect the cumulative exposure over 9 months of pregnancy. This nondifferential misclassification error tends to bias the association toward the null. Third, other pollutants emitted by quarries, such as PAHs, were not measured. It is well established that quarrying activities, particularly those involving rock crushing and blasting, can release significant quantities of heavy metals such as lead, cadmium, and arsenic into the surrounding environment. These metals can cross the placental barrier and have been associated with fetal growth restriction and LBW. Future studies should include measurements of these metals in both environmental and biological samples (maternal and cord blood) to better understand their contribution to the observed associations.

Our results align with biological mechanisms described in the literature. Van Den Hooven et al. demonstrated that prenatal exposure to PM2.5 was associated with reduced placental growth [7]. Nikasinovic described how PM2.5 induces epigenetic modifications in placental tissue [11]. Although cross‐sectional, our study provides local empirical data to fill the deficit identified by Glinianaia et al. in their systematic review on methodological gaps [14]. Green et al. also demonstrated that residential exposure to traffic was associated with an increased risk of spontaneous abortion [15].

The measured average concentrations ranged from 54.6 to 102.5 μg/m^3^, which is 5–20 times higher than the WHO 24‐h threshold of 15 μg/m^3^ [1]. These levels are also well above the WHO annual guideline of 5 μg/m^3^. These levels are comparable to those observed in other heavily polluted African metropolises [3]. The study by McFarlane et al. in Kinshasa had already reported an annual average of 43.5 μg/m^3^ [4]. The peaks between 4 AM and 8 AM coincide with blasting and crushing activities, confirming the industrial origin of the pollution.

After adjustment, gestational complications (hypertension, anemia, malnutrition, and diabetes) remained strongly associated with LBW: aOR = 4.67 [2.11–10.34], *p* < 0.001. This confirms data from the literature in the DRC. The association between gestational complications and LBW reflects the syndemic characteristic of limited‐resource contexts, where environmental and social vulnerabilities intersect [16]. In Lubumbashi, Luhete et al. also documented that maternal anemia and young age < 18 years were independent predictors of LBW [17]. Age < 18 years and primiparity were not significant after adjustment in our study, likely due to small sample sizes.

Our study collected data on maternal alcohol consumption, tobacco exposure, and nutritional factors as potential risk factors for LBW. None of these factors was significantly associated with LBW in our sample, which may be due to the relatively low prevalence of alcohol consumption (5.1%) and tobacco exposure (8.7%) in this population, as well as the limited variability in nutritional indicators. Nevertheless, these factors should be systematically assessed in future studies with larger sample sizes.

The main strength of this study is the combination of individual health data and local environmental measurements in a context where evidence is scarce in Sub‐Saharan Africa. In addition, the use of a validated calibration equation for the PurpleAir sensors ensures the accuracy of PM2.5 measurements in the specific context of Kinshasa. Although cross‐sectional, our study provides local empirical data to fill the deficit identified by Glinianaia et al. in their early systematic review on methodological gaps in pollution–fetal outcomes studies.

### 4.1. Limitations of the Study

Our study presents several limitations: The cross‐sectional design does not allow for establishing a causal relationship. Furthermore, a time lag exists between the births that occurred in 2021 and the PM2.5 measurements carried out in 2022. We hypothesized that the emission levels of the quarries were stable over this period.

The PM2.5 measurements over 48–72h per site do not represent prenatal exposure over 9 months and were conducted in September‐October 2022, the dry season, which could overestimate the annual average exposure. No individual exposure assessment was performed, and residential mobility during pregnancy was not evaluated. However, Bell and Belanger showed that more than 80% of pregnant women in low‐income urban contexts remain residentially stable during gestation [18]. The 75 μg/m^3^ threshold used to categorize exposure corresponds to the median of our measurements and has no established clinical value.

We were unable to measure or adjust for all potentially important factors: indoor air pollution related to cooking fuels, passive and active smoking (though we collected data on tobacco exposure, the small number of exposed women limited our statistical power), occupational exposures, detailed socio–economic indicators, and heavy metal exposure. These residual confounding biases may partly explain our associations. Future studies should include comprehensive exposure assessment, including personal monitoring, indoor air quality measurements, and biomarkers of exposure to heavy metals and other pollutants.

The sample size of certain subcategories, such as mothers < 18 years, *n* = 12, was small, limiting the power to detect significant associations. Despite these limitations, the consistency of the results with the international literature and the biological gradient by distance reinforce the internal validity of our conclusions.

## 5. Conclusion and Recommendations

This cross‐sectional study conducted in the Binza Ozone HZ shows that residing less than 500 m from a quarry is strongly associated with LBW, with a risk multiplied by 4 after adjusting for confounding factors. The prevalence of LBW (12.96%) is significantly higher than the provincial reference of 7.8% (MICS‐DRC 2017–2018), confirming the existence of a local aggravating factor. The measured PM2.5 concentrations were very high but were not significantly associated with LBW after adjustment.

These results suggest that proximity to quarries is a marker of a set of environmental and social exposures detrimental to pregnancy [13]. As Charles et al. pointed out, environmental exposure cannot be dissociated from the social determinants of health [19]. This environmental injustice is exacerbated in the DRC, where Bwana et al. have already documented multiple risk factors for LBW in contexts of socio–economic vulnerability in Kamina [20]. The potential role of heavy metals and other pollutants emitted by quarries warrants further investigation.

### 5.1. Recommendations

1. Short term: Awareness campaigns targeting pregnant women living near pollution sources, with advice to reduce exposure during industrial activity, peaks from 4 AM to 8 AM.
2. Medium term: Establishment of a national air quality monitoring network using validated low‐cost sensors [15]. Creation of a buffer zone of at least 1 km around quarries for new residential constructions. Integration of environmental risk assessment into prenatal care protocols [21]. Training of healthcare providers to systematically collect data on alcohol consumption, tobacco exposure, and nutritional status during ANC.
3. Long term: Development of a national regulatory framework for air quality aligned with WHO guidelines. Strengthening of environmental surveillance capacities at the provincial level. As recommended by UNEP for Sub‐Saharan Africa, “the development of national regulatory frameworks aligned with WHO guidelines, combined with investments in accessible surveillance systems, constitutes an absolute priority to protect vulnerable populations” [22].

Longitudinal studies with repeated and individual exposure measurements, including measurements of heavy metals and other pollutants, are necessary to confirm the specific role of PM2.5 and quantify the risk attributable to air pollution in the DRC. Doherty and Kinney emphasized that new global estimates of LBW should accelerate progress toward the sustainable development goals [23]. Our study provides local data to support these efforts in the DRC.

**Nomenclature**
PM2.5: Particulate matter with diameter ≤ 2.5 μm
LBW: Low birth weight (< 2500 g)
HA: Health area
HZ: Health zone
WHO: World Health Organization
DRC: Democratic Republic of the Congo
ANC: Antenatal care
FRM: Federal Reference Method
BAM: Beta Attenuation Monitor
aOR: Adjusted odds ratio
CI: Confidence interval

## Funding

No funding was received for this study.

## Conflicts of Interest

The authors declare no conflicts of interest.

## Data Availability

De‐identified data supporting the results of this study are available upon reasonable request addressed to the corresponding author.
